# Optogenetic modulation of descending prefrontocortical inputs to the dorsal raphe bidirectionally bias socioaffective choices after social defeat

**DOI:** 10.3389/fnbeh.2014.00043

**Published:** 2014-02-17

**Authors:** Collin Challis, Sheryl G. Beck, Olivier Berton

**Affiliations:** ^1^Department of Psychiatry, University of Pennsylvania Perelman School of MedicinePhiladelphia, PA, USA; ^2^Neuroscience Graduate Group, University of Pennsylvania Perelman School of MedicinePhiladelphia, PA, USA; ^3^Department of Anesthesiology, Children's Hospital of Philadelphia and University of Pennsylvania Perelman School of MedicinePhiladelphia, PA, USA

**Keywords:** dorsal raphe, ventromedial prefrontal cortex, serotonin, optogenetics, electrophysiology, depression and anxiety disorders, social perception, social defeat

## Abstract

It has been well established that modulating serotonin (5-HT) levels in humans and animals affects perception and response to social threats, however the circuit mechanisms that control 5-HT output during social interaction are not well understood. A better understanding of these systems could provide groundwork for more precise and efficient therapeutic interventions. Here we examined the organization and plasticity of microcircuits implicated in top-down control of 5-HT neurons in the dorsal raphe nucleus (DRN) by excitatory inputs from the ventromedial prefrontal cortex (vmPFC) and their role in social approach-avoidance decisions. We did this in the context of a social defeat model that induces a long lasting form of social aversion that is reversible by antidepressants. We first used viral tracing and *Cre*-dependent genetic identification of vmPFC glutamatergic synapses in the DRN to determine their topographic distribution in relation to 5-HT and GABAergic subregions and found that excitatory vmPFC projections primarily localized to GABA-rich areas of the DRN. We then used optogenetics in combination with *cFos* mapping and slice electrophysiology to establish the functional effects of repeatedly driving vmPFC inputs in DRN. We provide the first direct evidence that vmPFC axons drive synaptic activity and immediate early gene expression in genetically identified DRN GABA neurons through an AMPA receptor-dependent mechanism. In contrast, we did not detect vmPFC-driven synaptic activity in 5-HT neurons and *cFos* induction in 5-HT neurons was limited. Finally we show that optogenetically increasing or decreasing excitatory vmPFC input to the DRN during sensory exposure to an aggressor's cues enhances or diminishes avoidance bias, respectively. These results clarify the functional organization of vmPFC-DRN pathways and identify GABAergic neurons as a key cellular element filtering top-down vmPFC influences on affect-regulating 5-HT output.

## Introduction

The capacity to detect and interpret the affective state of others using non-verbal social cues (e.g., facial expression, vocal prosody, posture, body movement, and olfactory cues) is a necessary survival skill shared by many animal species (Chang et al., 2013; Oliveira, 2013). It allows individuals to anticipate harmful intentions of others and adapt through rapid approach or avoidance decisions (O'Connell and Hofmann, 2012). The capacity to conduct social-cognitive appraisal is also a determining aspect of human social competence (Todorov, 2008; Volman et al., 2011) and dysfunction of the neural systems that mediate socioaffective decisions are thought to contribute to excessive reassurance-seeking behaviors and social withdrawal, which are two symptomatic dimensions shared across several affective disorders, including major depression, and social phobia (Heuer et al., 2007; Seidel et al., 2010; Derntl et al., 2011; Stuhrmann et al., 2011; Cusi et al., 2012; Moser et al., 2012).

Serotonin (5-HT) is a neurotransmitter system that plays an evolutionarily conserved role in regulating affiliative and antagonistic behaviors (Canli and Lesch, 2007; Dayan and Huys, 2009; Rogers, 2011). Increases in 5-HT output, such as resulting from treatment with SSRI antidepressants, have consistently been shown to positively bias socioaffective appraisals and facilitate social affiliation and dominance in human and animals (Raleigh et al., 1991; Knutson et al., 1998; Tse and Bond, 2002; Bond, 2005; Harmer and Cowen, 2013). In contrast, 5-HT depletion facilitates socially defensive behaviors and aggression (Young and Leyton, 2002; Munafo et al., 2006). The fact that the output of ascending 5-HT neurons located in the dorsal raphe nucleus (DRN) is under top-down control by multiple forebrain areas (Peyron et al., 1998; Freedman et al., 2000; Chiba et al., 2001; Celada et al., 2002; Lee et al., 2003; Vertes, 2004) suggests a potentially key role for DRN afferent systems in the modulation of socioaffective responses. Studies conducted *in vivo* in anesthetized rodents combining electrical stimulation of the ventromedial prefrontal cortex (vmPFC) and extracellular recordings in the DRN demonstrated the rapid inhibition of putative 5-HT neurons (Varga et al., 2001; Celada et al., 2002). Parallel histological tracing studies demonstrated that DRN GABAergic neurons that are preferential targets of vmPFC projections could mediate the inhibitory responses recorded *in vivo* (Jankowski and Sesack, 2004). However, due to the limited specificity of electrophysiological signatures to predict neurochemical cell-type (Calizo et al., 2011), the identities of neuronal populations that compose the vmPFC-DRN microcircuit have not been fully elucidated. Furthermore, there is a lack of information about the possible topographical distribution of various DRN cellular populations thereby limiting the progress of studies assessing their causal role in socioaffective responses and other behaviors.

In recent studies we used a murine model of chronic social defeat stress (CSDS) that induces long lasting avoidance bias responsive to antidepressants to characterize the role of DRN microcircuits in the development and expression of social aversion (Espallergues et al., 2012; Challis et al., 2013; Crawford et al., 2013; Veerakumar et al., 2013). In mice susceptible to CSDS, but not in ones resilient, we detected a sustained sensitized synaptic inhibition of DRN 5-HT neurons, associated with a state of dramatically reduced intrinsic excitability of 5-HT neurons. Furthermore, we identified a subset of *GAD2*^+^ GABA neurons with sensitized excitatory synaptic input and intrinsic excitability, which monosynaptically inhibits nearby 5-HT neurons. Using optogenetic photo silencing we provided evidence of their key role in the associative process that underlie the development of social avoidance in susceptible mice (Challis et al., 2013). Interestingly, we noted that these sensitized GABAergic neurons appear to be located in circumscribed lateral subregions of the DRN heavily innervated by the vmPFC. These observations suggest a potentially unique role of inputs from the vmPFC in driving stress-induced plasticity of GABA neurons within the DRN that underlie the stabilization of avoidance bias after CSDS.

In the present study, we set out to test this hypothesis. We used *in vivo* optogenetics to drive or inhibit the synaptic inputs from vmPFC axons locally within the DRN during the sensory contact phase of CSDS. We also used viral tracing, whole-cell recordings, and optogenetic methods in slice preparations to further characterize the anatomical and functional organization of the vmPFC-DRN pathway. Our results directly show that excitatory projections from the vmPFC preferentially target and synaptically activate GABA neurons that are topographically distributed within the DRN. We also show that activation of these terminals paired temporally with exposure to social cues potentiates negative socioaffective bias and social avoidance, while inhibition of these inputs facilitates the maintenance of social engagement after defeat, a characteristic of resilient individuals. These results provide fundamentally novel insights about neural mechanisms implicated in the top-down control of 5-HT during socioaffective tasks and have important implications for the understanding and treatment of affective disorders.

## Materials and methods

### Animals

Eight- to twelve-week old male mice bred onto a C57BL/6 background were used for all experiments. Mice were housed on a 12-h light/dark cycle with food and water available *ad libitum*. All studies were conducted according to protocols approved by the University of Pennsylvania Institutional Animal Care and Use Committee. All procedures were performed in accordance with institutional guidelines. The large cohort of defeated mice used to determine social choice consisted of male C57 Black mice (*C57BL/6J*: JAX stock number 000664). Trained aggressor mice were retired CD-1 male breeder mice (*Crl:CD1*; Charles River Laboratories, Malvern, PA). To generate a mouse line with fluorescently labeled *GAD65*-containing GABAergic or serotonergic neurons, male knocking *GAD2-Cre* mice (*Gad*2^tm2(cre)Zjh^/*J*; JAX stock number 010802) (Taniguchi et al., 2011) or BAC transgenic *Pet1-Cre* mice (*B6.Cg-Tg(Fev-cre*)1Esd/J; JAX stock number 012712) (Scott et al., 2005) were respectively crossed to female floxed-stop controlled *tdTomato* (RFP variant) mice (*B6.Cg-Gt(ROSA)26Sor*^tm9(CAG−tdTomato)Hze^/*J*; JAX stock number 007908) (Madisen et al., 2010) to achieve fluorescent labeling of *Cre* containing cells. To achieve expression of optogenetic probes or fluorescent tracers in glutamatergic vmPFC neurons we used *CaMKIIa-Cre* mice (*B6.Cg-Tg(CamK2a-Cre)T29-1Stl/J*; JAX stock number 005359) (Tsien et al., 1996). With the exception of the CD-1 strain, all mice were procured from the Jackson Laboratory (Bar Harbor, ME).

### Virus and surgery

To express optogenetic or fluorescent proteins in glutamatergic neurons, adeno-associated virus (AAV) vectors were produced by and purchased from the University of Pennsylvania vector core (Philadelphia, PA) and injected into *CaMKIIa-Cre* mice. In this work we used AAVs for the *Cre*-inducible expression of the excitatory optogenetic probe *Channelrhodopsin* (AAV2/9.EF1a.DIO.hChR2(H134R)-EYFP.WPRE.hGH; Addgene #20298), inhibitory optogenetic probe *Archaerhodopsin* (AAV2/9.flex.CBA.Arch-GFP.W.SV40; Addgene #22222), fluorescent protein *tdTomato* (AAV2/1.CAG.FLEX.tdTomato.WPRE.bGH; Allen Institute #864) and *GFP* tagged Synaptophysin (AAV2/9.CMV.FLEX.Synaptophysin-Venus.WPRE.hGH; plasmid kindly provided by Anton Maximov, PhD, Department of Molecular and Cellular Neuroscience, The Scripps Research Institute). For stimulation of excitatory vmPFC terminals in the DRN of *GAD2-tdTomato* or *Pet1-tdTomato* mice we used an AAV for the *CaMKIIa*-driven expression of *Channelrhodopsin* fused to *YFP* (AAV2/9.CaMKII.ChR2-YFP.SV40; Stanford) (Mattis et al., 2012).

For viral injections, mice were anesthetized with isofluorane and stereotaxically injected unilaterally in the prelimbic region of the vmPFC (from Bregma, in mm: +1.8 AP, +0.8 ML, −2.7 DV, 15° angle) with 0.5 μl of virus. Viral yields (in GC) were 3.54 × 10^12^ for *ChR2-YFP*, 6.962 × 10^11^ for *Arch-GFP*, 2.049 × 10^12^ for *tdTomato* and 4.347 × 10^12^ for *CaMKIIa-ChR2-YFP*. Social defeat began 4 weeks post-surgery for non-cannulated mice to allow time for recovery and viral expression.

For *in vivo* optical stimulation, precut guide cannulae (Plastics One, Roanoke, VA) targeting the DRN (from Lambda, in mm: 0.0 AP, +0.8 ML, −3.3 DV, 15° angle) were secured to the skull using stainless steel skull screws and acrylic cement. A fitted dustcap dummy was secured atop the guide cannula and mice were placed back in homecages and allowed 6 weeks to recover. Body weight and behavior was monitored during recovery. Three days before the start of experiment, a homemade fiber optic with ferrule connector (described below) was inserted into the guide cannula and secured with acrylic cement.

### Preparation of optical fibers

A Two hundred μm core, 0.37 NA standard multimode fiber (Thorlabs, Newton, NJ) was stripped of cladding, passed through a 230 μm multimode ceramic zirconia ferrule (Precision Fiber Products, Milpitas, CA), and secured in place using fiber optic connector epoxy (Fiber Instrument Sales, Oriskany, NY). Ferrules were then polished and cut to length to target the DRN. They were tested for light output and sterilized with 70% ethanol.

### Chronic social defeat stress

We use a modified chronic social defeat stress (CSDS) paradigm to induce social avoidance (Golden et al., 2011; Challis et al., 2013). Our model consists of exposing male mice to alternating periods of physical contact with a trained CD1 aggressor male mouse (5 min) and protected sensory contact via separation by a perforated Plexiglass partition (20 min) before returning to home cages overnight. The 20 min of sensory contact is sufficient to induce a significant decrease in social interaction compared to undefeated mice or mice that were not exposed to a sensory period after physical contact. This effect has been previously described Challis et al. (2013). This continued for 10 consecutive days with exposure to a novel aggressor each day. Control animals were also singly housed and were only exposed to daily sensory contact with novel aggressors. On day 11, social approach or avoidance behavior toward an unfamiliar CD1 social target was assessed in a two-trial social interaction task. In the first 2.5-min trial (“no target”), experimental mice explored a dimly lit (55 lux) open-field arena containing an empty wire mesh cage on one edge of the arena (see Figure 6A). In the second 2.5-min trial (“target present”), experimental mice were reintroduced to the arena now with an unfamiliar CD1 aggressor positioned in the mesh cage. TopScan video tracking software (CleverSys, Reston, VA) was used to measure the time spent in the interaction zone surrounding the target box.

### Immunohistochemistry

Animals were transcardially perfused with 4% paraformaldehyde and brains were processed for standard single or dual immunolabeling methods as previously described (Espallergues et al., 2012). For detection of *cFos*, we used an affinity purified rabbit polyclonal antibody raised against the N-terminus of human *cFos* (1:1000 dilution; SC-52, Santa Cruz Biotechnology, Santa Cruz, CA). To enhance *GFP* expression we used a chicken anti-*GFP* antibody (1:1000 dilution; GFP-1020, Aves Labs, Inc., Tigard, OR). Primary antibodies were detected using fluorescent secondary antibodies obtained from Jackson ImmunoResearch Laboratories (1:500 dilution; West Grove, PA).

### Cell counting

To map neuronal populations in the DRN, 30 μm serial sections of the DRN were collected every 120 μm between −4.36 mm and −4.96 mm from Bregma. Native *tdTomato* fluorescence and immuno-enhanced *GFP* fluorescence of *SynP* labeled vmPFC terminals were visualized using confocal microscopy. Slices from corresponding rostro-caudal levels between mice were aligned on a map based on location of the aqueduct. Neurons and terminals were manually drawn for each level of the DRN.

To quantify *cFos* colocalization with *tdTomato*^+^ neurons, slices were stained for *cFos* and labeled neurons were manually counted in the DRN of each section. Colocalization with *tdTomato* was defined as nuclear localization of the *cFos* signal and was manually counted by an experimenter blind to the experimental condition of the mice from which the slices originated. There was not a significant variation of total number of *tdTomato*^+^ cells within each strain.

To determine whether spatial distribution of synaptic vmPFC inputs traced using *SynP-GFP* correlated with the distribution of *GAD2-tdTomato* or *Pet1-tdTomato* neurons, we divided corresponding coronal views of the DRN in *GAD2-tdTomato*, *Pet1-tdTomato* and *SynP-GFP* injected *CaMKIIa-Cre* mice into 10 × 10 grids and tested for correlations between *SynP-GFP* and *tdTomato* fluorescence across the grid. This was done at each of the 6 rostro-caudal levels across the DRN. Fluorescent intensity within each grid box was calculated using the ImageJ “Measure” function which converts red, green, and blue (RGB) pixel values to brightness using the formula V = (R + G + B)/3. These intensity values were then normalized to the grid box with the highest intensity. Correlations were tested using the Pearson coefficient and plotted using linear regression.

### Electrophysiology

Brain slices were prepared as previously described (Crawford et al., 2010, 2013; Calizo et al., 2011; Espallergues et al., 2012; Challis et al., 2013; Howerton et al., 2013). The 200 μm coronal slices containing DRN were placed in aCSF (in mM, NaCl 124, KCl 2.5, NaH_2_PO_4_ 1.25, MgSO_4_ 2.0, CaCl_2_ 2.5, dextrose 10, NaHCO_3_ 26) at 37°C, aerated with 95% O_2_/5% CO_2_. After 1 h, slices were kept at room temperature. Tryptophan (2.5 mM) was included in the holding chamber to maintain 5-HT synthesis, but was not in the aCSF perfusing the slice in the recording chamber. Individual slices were placed in a recording chamber (Warner Instruments, Hamden, CT) and perfused with aCSF at 2 ml/min maintained at 32°C by an in-line solution heater (TC-324, Warner Instruments). Neurons were visualized using a Nikon E600 upright microscope fitted with a 60X water immersion objective and targeted under DIC or fluorescent filters. Resistance of electrodes was about 8–10 MOhms when filled with a recording solution composed of (in mM) K-gluconate (130), NaCl (5), Na phosphocreatine (10), MgCl_2_ (1), EGTA (0.02), HEPES (10), MgATP (2) and Na_2_GTP (0.5) with 0.1% biocytin and a pH of 7.3. Whole-cell recordings were obtained using a Multiclamp 700 B amplifier (Molecular Devices, Sunnyvale, CA). Cell characteristics were recorded using current clamp techniques as previously described (Crawford et al., 2010; Espallergues et al., 2012). Signals were collected and stored using Digidata 1320 analog-to-digital converter and pClamp 9.0 software (Molecular Devices). Collection of EPSC data was as previously described (Crawford et al., 2011) and performed with bath application of 20 μM bicuculline to block GABA synaptic activity. To characterize light-evoked ESPC activity, 20 μM DNQX was applied to the bath to block AMPA receptor activity. All drugs were made in stock solutions, diluted on the day of the experiment, and added directly to the ACSF.

### Electrophysiology data analysis

Synaptic properties were analyzed using MiniAnalysis (Synaptosoft, Decatur, GA) as previously described (Crawford et al., 2011, 2013). Synaptic events were analyzed using parameters optimized for each cell with the detection threshold set beyond the maximum values of the all-points noise histogram for a portion of the trace containing no detectable synaptic events. This threshold generally ranged from 5 to 8 pA. MiniAnalysis generates a summary table containing the mean and median values for the frequency, amplitude, rise time (10–90%), decay time, and event half width (50%). For each cell, at least 200 events were chosen at random and manually filtered to exclude multiple peaks then combined to obtain an averaged EPSC or IPSC for each cell to obtain values for decay time, event area, and event time half-width. Additional statistical analysis is described below. Data reported are means ± s.e.m.

### Optical stimulation

For *in vivo* stimulation, mice with previously implanted fiber optic ferrules were connected to a 200 μm, 0.37 NA patch cord via zirconia sleeve that was then connected to a diode-pumped solid-state (DPSS) laser through an FC/PC adaptor and rotary joint. We used blue (473 nm, BL-473-00100-CWM-SD-05-LED-0) and yellow (561 nm, GR-561-00100-CWM-SD-05-LED-F) DPSS lasers obtained from OEM Laser Systems (Bluffdale, UT). Power output was measured using an optical sensor (Thorlabs, Newton, NJ) to be about 10 mW. Intensity was calculated using a model predicting irradiance in mammalian tissues (http://www.stanford.edu/group/dlab/cgi-bin/graph/chart.php). From a 200 μm fiber optic tip, estimated intensity was 7.33 mW mm^−2^ for blue laser stimulation and 7.05 mW mm^−2^ for yellow laser stimulation. For stimulation of vmPFC terminals expressing *ChR2* to determine DRN neuronal activation, the day before the stimulation mice were connected to the laser and housed in home cages overnight. The following day we performed sustained blue light stimulation at 25 Hz with 10 ms pulse width for 20 min without disturbing the mouse. For stimulation of *ChR2* during CSDS mice were connected to the laser after physical defeat and we performed sustained blue light stimulation at 25 Hz and 10 ms pulse width during 20 min of sensory contact. For stimulation of *Arch* during CSDS we performed constant yellow light stimulation for 20 min.

For stimulation of brain slices expressing *ChR2* in vmPFC terminals, a prepared 200 μm core, 0.37 NA standard multimode fiber was lowered into the recording chamber and submerged below ACSF. The tip of the fiber was positioned approximately 1 mm from the vmPFC or DRN, illuminating the entire region. Stimulation of the DRN was either performed at 0.5 Hz with a 10 ms pulse width for an 8 s epoch with 22 s between sweeps or at 25 Hz with a 5 ms pulse width for a 20 s epoch with 10 s between sweeps. Stimulation of the vmPFC was performed at either 5, 25, or 100 Hz with a 5 ms pulse width for a 2 s epoch with 18 s between sweeps. Laser intensity was estimated to be 18.07 mW mm-2.

### Data analysis and statistics

For multiple group comparisons, all variables were distributed normally based on Bartlett's test and analyzed using parametric statistics (i.e., One-, Two-Way ANOVAs, between group or with repeated measures, followed by Fisher's PLSD test where appropriate). Comparisons between two groups were performed using Student's *t*-test. Statistical analysis was performed using Statistica (StatSoft, Tulsa, OK). To calculate spatial correlation between *SynP* and *tdTomato* fluorescence, the Pearson correlation coefficient (Pearson's r) was calculated. To determine rate of cumulative time spent per second, the slope of the linear regression and goodness of fit (*r*^2^) was calculated. Statistical significance was defined as a *p* value < 0.05. All data are presented as the mean ± s.e.m. Outlying values (3 standard deviations from the mean) were excluded from group means.

## Results

### Excitatory vmPFC terminals and GABAergic neurons in the DRN have overlapping topographic distributions

**Figure 1:**
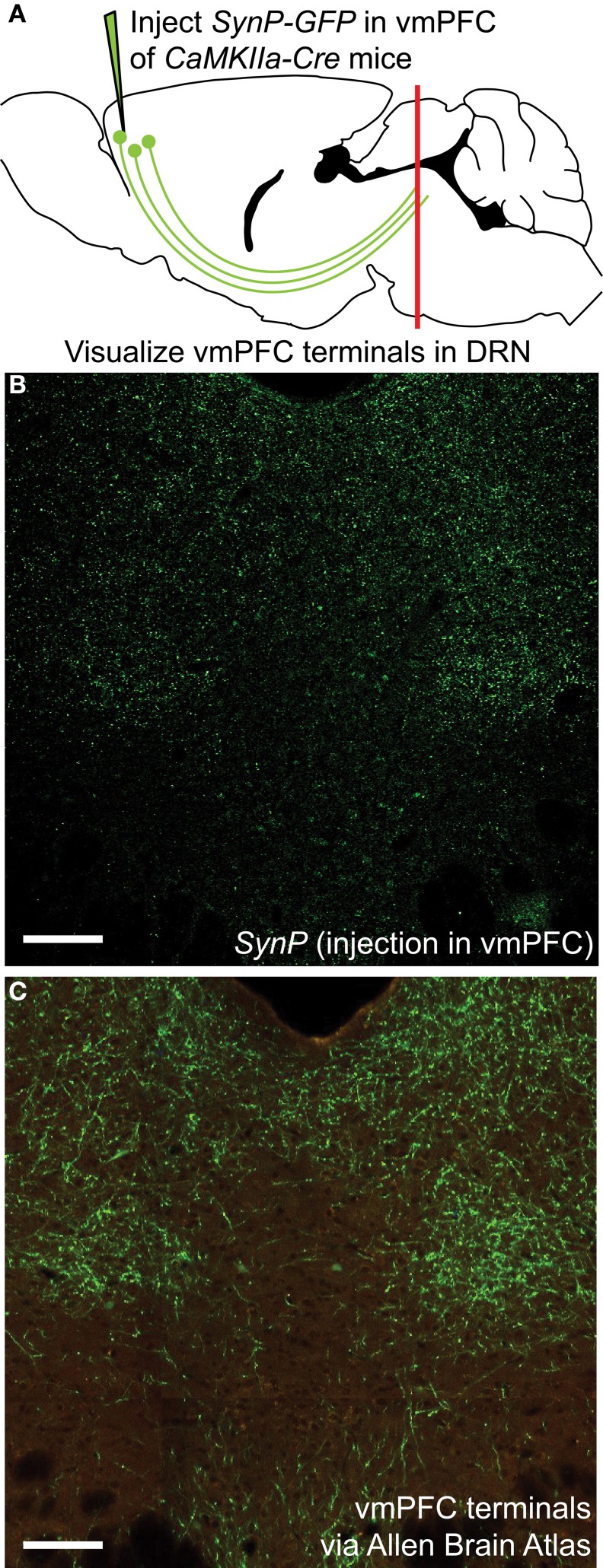
**Visualization of vmPFC axon terminals in the DRN (A)** An AAV vector coding for the *Cre*-dependent expression of the fluorescently tagged synaptic protein *Synaptophysin* (*SynP-GFP*) was injected in the vmPFC of *CaMKIIa-Cre* mice. Topographic distribution of vmPFC axon terminals in the DRN was visualized using confocal microscopy. **(B)** Distribution of vmPFC terminals as determined by *SynP-GFP* expression as reported in this study. Scale bar 50 μm. **(C)** Anterograde tracing using an AAV-GFP vector injected in the vmPFC as reported by the Allen Brain Connectivity Atlas. Picture is courtesy of the Allen Mouse Brain Atlas [Internet]. © 2012 Allen Institute for Brain Science (Seattle, WA). Available from: http://mouse.brain-map.org. Despite use of different tracing methods, the pattern of innervation revealed is strikingly similar, with sparse innervation of the midline area of the DRN, classically containing 5-HT neurons, and denser innervation of lateral portions of the DRN. Scale bar 50 μm.

**Figure 2:**
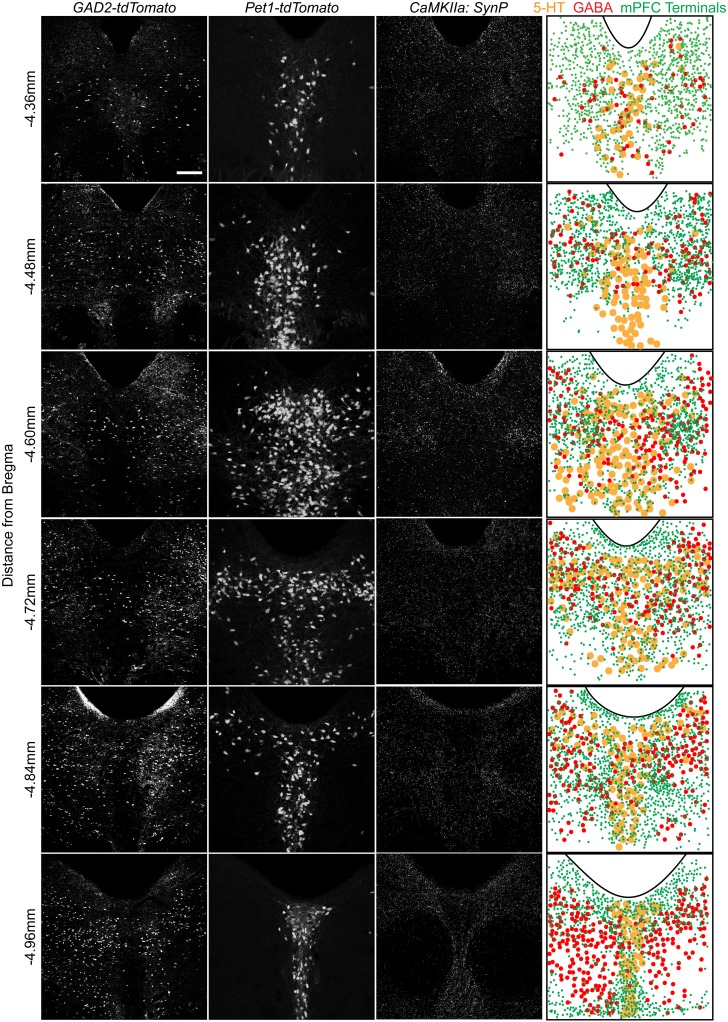
**Spatial organization of 5-HT and GABA neurons and vmPFC terminals in the DRN**. Native fluorescence of GABA (*GAD2-tdTomato*, column 1) and 5-HT (*Pet1-tdTomato*, column 2) neurons as well as antibody enhanced glutamatergic vmPFC terminal fluorescence (*CaMKIIa*: *SynP-GFP*) is visualized in serial sections of the DRN. Individual cellular or synaptic localization was overlaid on a map using the aqueduct as a reference. Scale bar 50 μm.

**Figure 3:**
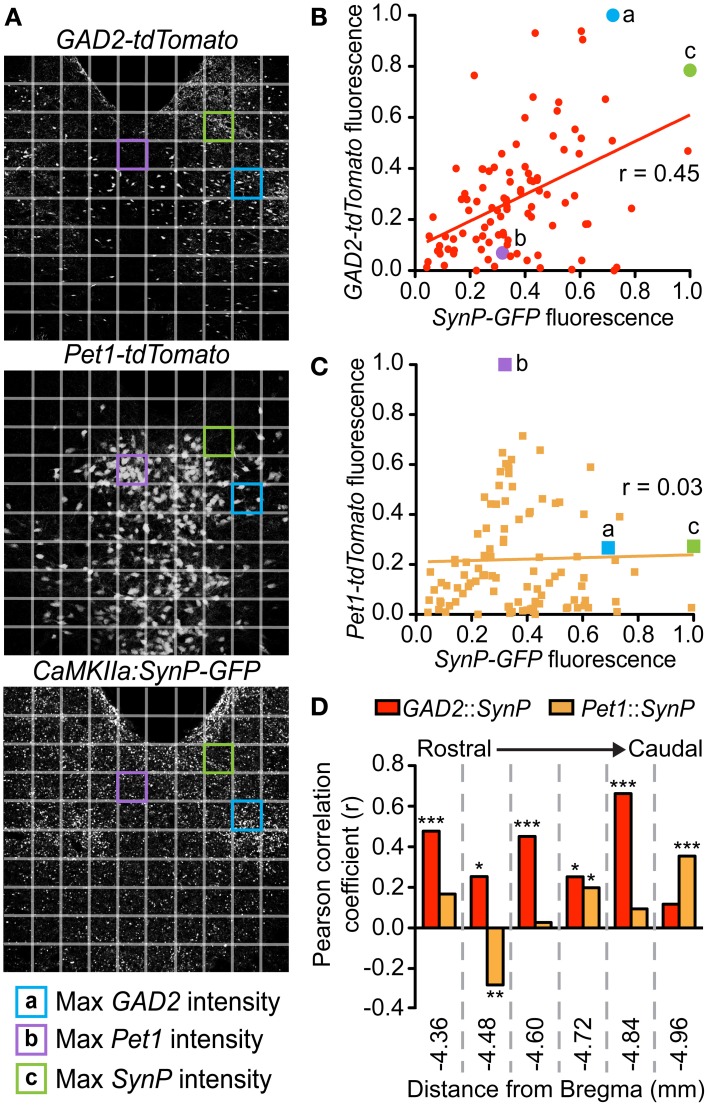
**Topographic distribution of vmPFC terminals correlates with GABAergic populations in the DRN**. At each of the 6 rostrocaudal levels of the DRN, images of each fluorescent signal were divided into a 10 × 10 grid. Relative intensities were calculated for each of the *SynP-GFP* (vmPFC terminals), *GAD2-tdTomato* (GABA neurons) and *Pet1-tdTomato* (5-HT neurons) fluorescent signals for each box of the grid at every rostrocaudal level. **(A)** For example, depicted here are DRN slices at −4.60 mm from Bregma. Highlighted grid boxes depict areas that were calculated to have the highest (a) *GAD2-tdTomato* intensity, (b) *Pet1-tdTomato* intensity and (c) *SynP-GFP* intensity. Intensity values of *SynP-GFP* were correlated with that of **(B)** *GAD2-tdTomato* or **(C)** *Pet1-tdTomato* at each of the 6 rostrocaudal levels. These were graphed on scatter plots with (x,y) coordinates plotted as (*SynP-GFP* intensity, *tdTomato* intensity). Grid boxes highlighted in **(A)** are plotted in **(B)** and **(C)** as examples. Pearson's correlation coefficients (r) were calculated for *GAD2-tdTomato* or *Pet1-tdTomato* vs. *SynP-GFP* with *r* = 1 signifying strong correlation, *r* = 0 signifying no correlation and *r* = −1 signifying strong negative correlation. **(D)** Overall there is a stronger correlation of topographic distribution of GABA neurons with vmPFC terminals than 5-HT neurons except in the most caudal DRN. (Bars with asterisks indicate Pearson coefficients that are significantly non-zero; ^*^p < 0.05, ^**^p < 0.005, ^***^p < 0.001).

To assess the distribution of vmPFC axon terminals in the DRN we performed viral mediated tracing using a *Cre*-dependent AAV vector coding for a *GFP*-tagged variant of the synaptic protein *Synaptophysin* (*SynP*) (Veerakumar et al., 2013). To selectively target excitatory neurons in the vmPFC (Lee et al., 2003; Commons et al., 2005) the vector was injected in male mice of the *CaMKIIa-Cre* line (Calhoun et al., 1996) (Figure 1A). We then assessed the distribution of excitatory vmPFC terminals by visualizing *SynP-GFP* fluorescence in the DRN (Figure 1B). The distribution pattern of vmPFC terminals shows a striking similarity to images from the Allen Brain Connectivity Atlas after injetion of an AAV expressing *EGFP* in the vmPFC (Figure 1C). To determine whether synapses formed by these terminals occur preferentially in areas enriched in 5-HT or GABA neuron subtypes, we compared the topographic distribution of *SynP-GFP* punctas with that of genetically labeled GABA (*GAD2-tdTomato*) or 5-HT (*Pet1-tdTomato*) neurons at similar rostro-caudal levels (Challis et al., 2013) (Figure 2). We found that GABA neurons tended to be primarily distributed in the lateral aspects of the DRN, while 5-HT neurons were concentrated in the midline in the anterior and posterior DRN and were in the midline as well as branched to the dorsolateral DRN, or lateral wings (Crawford et al., 2010), in the mid DRN. Glutamatergic vmPFC terminals on the other hand clustered in the dorsolateral and ventrolateral DRN in the anterior to mid DRN before gathering in the dorsomedial and ventromedial DRN of the most posterior slices. We compared the relative fluorescent intensity of *SynP-GFP* with the intensities of *GAD2-tdTomato* or *Pet1-tdTomato* signals to determine if there was a topographic correlation in the DRN (Figure 3). Scatter plots summarize the correlation found *SynP-GFP* intensity and either *GAD2-tdTomato* (Figure 3B) or *Pet1-tdTomato* (Figure 3C) intensity. We found that throughout the DRN, distribution of vmPFC terminals correlated more strongly with GABA neurons than with 5-HT neurons except in the most caudal extent of the DRN as determined by calculation of Pearson correlation coefficients [Number of mice (slices per mouse) = 3(6); Figure 3D].

### Descending excitatory projections from the vmPFC preferentially drive DRN *cFos* induction in GABAergic neurons

**Figure 4:**
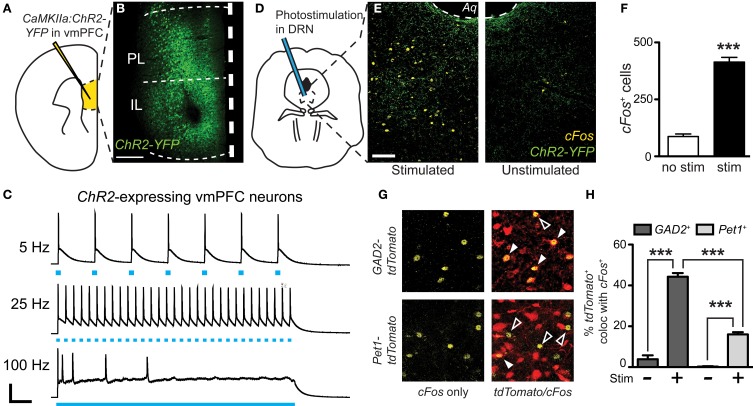
**Preferential *cFos* induction in GABA neurons after photostimulation of vmPFC terminals in the DRN. (A)** *CaMKIIa*-driven *ChR2-YFP* was injected in the vmPFC of *GAD2-tdTomato* or *Pet1-tdTomato* mice. **(B)** After allowing 6 weeks, robust expression of *ChR2-YFP* was achieved. Scale bar 25 μm. **(C)** Fluorescent neurons in layer V of the vmPFC were recorded. Current-clamp traces of *YFP*^+^ vmPFC neurons demonstrate temporal fidelity of *ChR2*-mediated photocurrents at 5 and 25 Hz, and loss of precision at 100 Hz. Scale bar 40 pA, 0.2 s. **(D)** At the same time point post-injection (6 weeks), vmPFC terminals in the DRN were photostimulated for 20 min using 473 nm light (10 mW, 25 Hz, 10 ms pulse width). **(E)** *cFos* immunofluorescence was evaluated after photostimulation to indicate neuronal activation. Scale bar 50 μm. **(F)** Photostimulation resulted in a significant increase in total *cFos* expression with no difference between genotype (Student's *t*-test, ^***^p < 0.001). **(G)** Quantification of *cFos* colocalization with native *tdTomato* fluorescence was used to determine the neuronal populations activated. Closed arrows indicate colocalized neurons. **(H)** Without laser stimulation there is minimal *cFos* expression. After photoactivation of vmPFC terminals there were increases in neuronal activation of both GABA and 5-HT neurons, however a much higher percentage of *GAD2-tdTomato* neurons colocalize with *cFos* (Two-Way ANOVA, Fisher *post-hoc*, ^***^p < 0.001).

Using immediate early gene mapping, we previously established that exposure to CSDS activates DRN GABA neurons preferentially over 5-HT neurons and that the topographic distribution of these neurons overlaps with that of vmPFC terminals (Challis et al., 2013). Here, we tested whether direct activation of the terminals would increase *cFos* primarily in GABA neurons. We did this by stereotaxic infusion of an AAV vector leading to *CaMKIIa*-driven expression of *YFP*-tagged *Channelrhodopsin-2* (*ChR2-YFP*) in the vmPFC (Ji and Neugebauer, 2012) (Figures 4A,B). Previous studies have shown that this approach restricts expression chiefly to pyramidal neurons (Tsien et al., 1996). Twenty-eight days after surgery we observed robust expression of *ChR2-YFP* in the vmPFC that spread through infralimbic (IL) and prelimbic (PL) regions. We confirmed the expression and function of *ChR2* in the vmPFC by performing current-clamp recordings of *YFP*^+^ neurons during exposure to trains of pulsed light (Figure 4C). Photostimulation frequencies from 5 Hz up to 25 Hz resulted in pulse-locked action potentials, however at 100 Hz, a stimulation frequency similar to that of deep brain stimulation (DBS), this fidelity was lost. To then stimulate terminals directly in the DRN, we implanted cannulae targeting the DRN 3 weeks after injection (Figure 4D). Three days before stimulation fiber optic ferrules were inserted in the cannulae and secured to the skull. The day prior to testing, mice were connected to the laser via fiber optic patch cable and remained isolated in home cages overnight. On the day of testing we performed laser stimulation without disturbing the mice to prevent activation by handling. We used a selective photoexcitation protocol of vmPFC axon terminals in the DRN similar to an approach that has previously been shown to produce robust time-locked behavioral effects dependent on the resulting local release of glutamate in the DRN (Warden et al., 2012). Here, photostimulation of the vmPFC terminals in the DRN for 20 min (473 nm, 10 mW, 25 Hz, 10 ms pulse width) resulted in a significant overall increase in *cFos* expression compared to unstimulated controls [Student's *t-test*, *t*_(10)_ = 14.89, *p* < 0.001; *n* = 6–8 per group; Figures 4E,F]. In *GAD2-tdTomato* and *Pet1-tdTomato* mice, this stimulation protocol led to significantly higher activation of *GAD2*- over *Pet1*-labeled neurons [Two-Way ANOVA, genotype × stim, *F*_(3, 13)_ = 102.07, *p* < 0.001, Figures 4G,H]. Control mice that were connected to the laser, but not stimulated did not display an increase in *cFos* immunoreactivity. Mice that were injected with sham virus also did not display an increase in *cFos* expression (data not shown). This outcome, in line with previous neuroanatomical and ultrastructural data, implicates GABAergic neurons as the primary postsynaptic targets of vmPFC afferents in the DRN.

### Photostimulation of vmPFC terminals in DRN drives timed-locked AMPA-mediated postsynaptic responses in GABAergic but not 5-HT neurons

**Figure 5:**
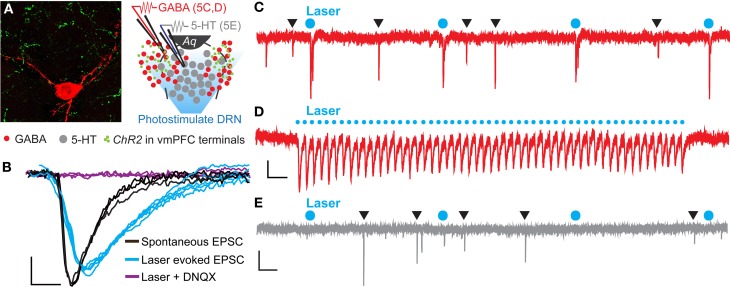
**Photoactivation of vmPFC terminals results in time-locked EPSC events in DRN GABA neurons. (A)** High magnification confocal image of a biocytin-filled recorded *GAD2-tdTomato* neuron (red) in close contact with axon terminals from vmPFC neurons (green) anterogradely traced using AAV-*CaMKIIa*-*ChR2-YFP*. Inset displays a schematic of DRN topography in recorded slices from *GAD2-tdTomato* mice. Recorded neurons were purposely selected in ventrolateral subregions of the DRN that are rich in afferents from the vmPFC while the DRN was photostimulated. *Aq*—aqueduct. **(B)** Average voltage-clamp traces of spontaneous and laser evoked EPSC events. Application of 20 μM DNQX prevented laser-evoked EPSC events. Scale bar 4 pA, 3 ms. **(C)** Raw voltage-clamp data trace recorded from a *GAD2-tdTomato* neuron during pulsed photoactivation of *ChR2* containing vmPFC terminals (473 nm, 10 mW, 0.5 Hz, 10 ms pulse width). Blue circles mark laser pulses. Black triangles indicate spontaneous EPSC events. Scale bar 10 pA, 0.5 s. **(D)** EPSC events were time-locked to a 25 Hz laser stimulation train. Scale bar 5 pA, 0.1 s. **(E)** Recordings from *Pet1-tdTomato* neurons did not display EPSC events in response to laser stimulation. Scale bar 10 pA, 0.5 s.

To determine if the vmPFC drives synaptic activity of GABA neurons in the DRN we again injected *CaMKIIa*-driven *ChR2* into the vmPFC of *GAD2-tdTomato* mice. After 6 weeks, we then prepared slices of the DRN for whole-cell patch clamp electrophysiology and recorded from genetically labeled *GAD2*^+^ GABA neurons (Figure 5A). Brief pulses of 473 nm laser stimulation (0.5 Hz, 10 mW, 10 ms pulse width) resulted in pulse-locked EPSC events (Figure 5C) that remained in high fidelity up to 25 Hz (Figure 5D). In the presence of DNQX these events disappeared, indicating that the recorded excitatory events were mediated by AMPA receptors (Figure 5B). Comparing laser-evoked EPSCs to spontaneous events revealed significant differences in event rise time [Student's *t*-test, *t*_(12)_ = 4.56, *p* < 0.001; number of mice (number of neurons) = 2(12)] and decay time [Student's *t*-test, *t*_(12)_ = 2.16, *p* < 0.05] and trends toward significance in event amplitude and charge transfer (Figure 5B and Table 1). These differences indicated that the photostimulation of vmPFC fibers resulted in a unique postsynaptic response that was distinguishable from spontaneous quantal release. Using these stimulation parameters we were able to record postsynaptic responses in 25% of the recorded GABA neurons (12 total neurons). In contrast, recording from identified 5-HT neurons in *Pet1-tdTomato* mice did not yield any stimulated postsynaptic responses (12 total neurons; Figure 5E). These results reinforce the premise that the vmPFC sends glutamatergic projections directly to GABAergic neurons in the DRN.

**Table 1:** **Comparison of spontaneous vs. laser-evoked EPSC events in DRN GABA neurons**.

	**Rise (ms)**	**Amplitude (pA)**	**Decay (ms)**	**Area under curve (pA)**
Spontaneous	1.92 ± 0.15	14.68 ± 1.61	3.74 ± 0.57	−57.68 ± 6.56
Laser-evoked	2.84 ± 0.14^***^	11.55 ± 1.43	4.66 ± 0.13^*^	−67.81 ± 9.37

*Data are expressed as mean values ± s.e.m*.

* *p < 0.05*,

*** *p < 0.001 values different from spontaneous EPSC events as determined by unpaired t-test*.

### Photoactivation and photoinhibition of vmPFC terminals in the DRN during post-defeat sensory contact period has opposite effects on avoidance behavior

**Figure 6:**
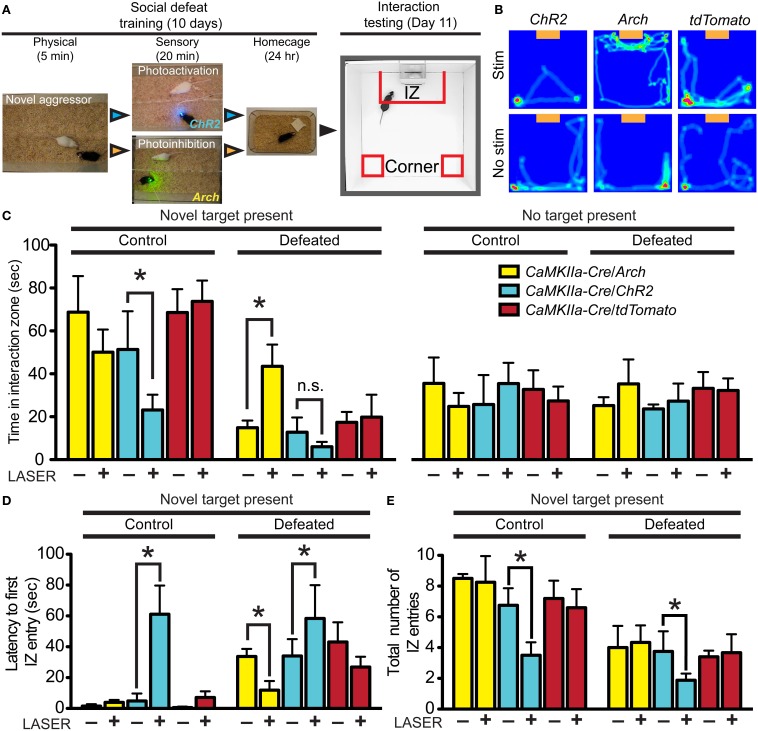
**Divergent effects of vmPFC terminal photostimulation and photoinhibition in the DRN. (A)** *CaMKIIa-Cre* mice were injected in the vmPFC with *Cre*-dependent AAVs coding for the expression of *ChR2-YFP*, *Arch-GFP* or *tdTomato*. Cohorts of mice were exposed to 5 min of physical defeat followed by 20 min of sensory contact with either photoactivation by *ChR2* (473 nm, 10 mW, 25 Hz, 10 ms pulse width) or photoinhibition by *Arch* (561 nm, 10 mW) of vmPFC terminals via implanted fiber optic targeting the DRN. Mice were then placed in homecages overnight. This was repeated with exposure to a novel aggressor each day. On day 11, mice were assessed for approach or avoidance in the social interaction test. *IZ*—interaction zone. **(B)** Heat maps depicting representative behavioral effects of photoactivating (C*hR2*) or photoinhibiting (*Arch*) vmPFC terminals during the sensory period of social defeat on interaction with a novel social target (orange box). Red and green areas depict areas where mice spent the most time. No effect was observed in mice injected with sham *tdTomato* virus. **(C)** In defeated mice, photosilencing of vmPFC terminals prevented a decrease in social interaction (Two-Way ANOVA, Fisher *post-hoc*, ^*^p = 0.050) while photoactivation did not decrease interaction times significantly from *ChR2*-expressing mice that did not receive laser stimulation. Undefeated control mice whose vmPFC terminals in the DRN were stimulated displayed a significant decrease in social interaction compared to unstimulated counterparts (Two-Way ANOVA, Fisher *post-hoc*, ^*^p = 0.049). No effect of virus of photostimulation was observed when a novel social target was not present. **(D)** With a novel social target present, mice whose vmPFC terminals were photoactivated via *ChR2* during sensory contact displayed significant increases in latencies to first enter the IZ in both control (Two-Way ANOVA, Fisher *post-hoc*, ^*^p = 0.033) and defeated conditions (^*^p = 0.049). Defeated mice whose vmPFC terminals in the DRN were photosilenced via *Arch* during sensory contact displayed shorter latencies to first enter the IZ (^*^p = 0.048). **(E)** Total number of entries into the IZ was decreased in both control and defeated mice whose vmPFC terminals in the DRN were photoactivated during sensory contact (Two-Way ANOVA, Fisher *post-hoc*, control ^*^p = 0.042, defeated ^*^p = 0.049).

We have previously demonstrated that inhibition of DRN GABAergic neurons prevents the acquisition of social avoidance after defeat, but did not change expression of an already acquired avoidance phenotype (Challis et al., 2013). To determine whether vmPFC terminals that drive GABA neurons' activity in the DRN also contribute to the encoding of social aversion, we expressed optogenetic probes in *CaMKIIa-Cre* neurons in the vmPFC and photostimulated or photoinhibited terminals directly in the DRN. To activate glutamatergic vmPFC projections, we used *ChR2* (473 nm) and to inhibit we expressed *Archaerhodopsin* (*Arch*, 543 nm). Because we have previously shown that a period of 20 min of post-defeat sensory exposure is necessary and sufficient to trigger a significant avoidance response (Challis et al., 2013), mice were connected to the laser via fiber optic connector and stimulated daily during this period before returning to home cages overnight (Figure 6A). This was repeated for 10 days with exposure to a novel CD1 aggressor mouse every day. On day 11, approach-avoidance choices were evaluated by performing the social interaction test using a novel social target (Figure 6B).

Mice from the control group injected with a sham vector and receiving laser stimulation in the DRN displayed interaction times similar to that previously reported in defeated naïve mice indicating that the cannulation and potential thermal artifacts caused by laser manipulation, do not *per se* significantly alter the development of social avoidance (Challis et al., 2013) (Figure 6C). In contrast, mice whose vmPFC terminals were photoinhibited in the DRN did not display typical social avoidance and maintained high levels of approach during social interaction testing [Two-Way ANOVA, virus × stim, *F*_(11, 50)_ = 6.58, *p* < 0.001; *n* = 6–10 mice per group]. On the other hand, defeated mice whose vmPFC terminals were photoactivated tended to show reductions in social interaction compared to mice injected with sham virus, although this difference did not reach statistical significance due to a floor effect on the expression of social avoidance. Interestingly, control mice that did not undergo defeat, but received photoactivation of vmPFC terminals in the DRN in the presence of a CD1 social target also subsequently displayed a significant decrease in time spent [Two-Way ANOVA, virus × stim, *F*_(11, 50)_ = 4.50, *p* = 0.002; Figure 6D] and in total entries in the social interaction zone [Two-Way ANOVA, virus × stim, *F*_(11, 50)_ = 6.85, *p* < 0.001; Figure 6E].

### Increased vmPFC drive of DRN delays decision to approach novel social target

**Figure 7:**
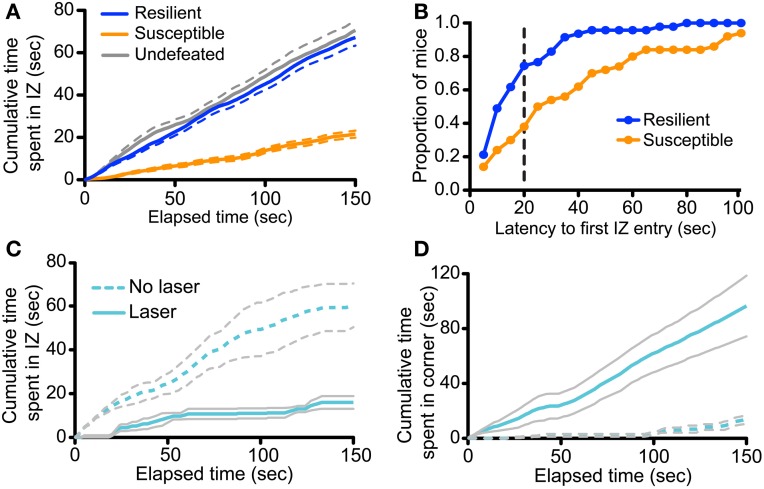
**Defeat and photoactivation of vmPFC terminals in the DRN bias social approach/avoidance choices. (A)** Analysis was performed on 97 previously defeated and 20 undefeated mice. Defeated mice were characterized as resilient or susceptible based on previously described criteria (Golden et al., 2011; Challis et al., 2013). The cumulative time spent in the interaction zone (IZ) was plotted across the duration of the social interaction test. The cumulative interaction time of susceptible mice significantly diverged from that of both resilient and control mice at 4 s (Repeated measures ANOVA, Fisher *post-hoc*, *p* = 0.020 at 4 s). Data shown is average per group. Dashed lines represent the s.e.m. **(B)** Latencies to the first IZ entry were determined in 5-s bins. Plotted are the proportions of mice within each behavioral group to display first IZ entry within the given timeframe or less. A greater proportion of resilient mice first entered the IZ faster that of susceptible mice (Kolmogorov-Smirnov test, *p* < 0.001). The dashed vertical line represents the latency at which the proportion discrepancy between the two behavioral is the largest (0.36). **(C)** Cumulative time spent in the IZ is plotted across time in the social interaction test for undefeated *CaMKIIa-Cre* mice expressing *ChR2* in the vmPFC with and without laser stimulation of vmPFC terminals in the DRN. Increase in cumulative interaction time from 0 s did not significantly differ until 60 s in laser-treated mice (Repeated measures ANOVA, Fisher *post-hoc* within Laser group, *p* = 0.045 at 60 s). **(D)** Cumulative time spent in the corners distal to the novel social target is plotted across time in the social interaction test. Increase in cumulative corner time from 0 s significantly differed at 14 s in laser-treated mice (Repeated measures ANOVA, Fisher *post-hoc* within Laser group, *p* = 0.047 at 14 s).

To gain further insight into how manipulation of vmPFC-DRN during CSDS training alters subsequent avoidance behaviors, we examined the effect of this manipulation on the time-course of social approach-avoidance behaviors during the interaction test. We first characterized the temporal distribution of the bouts of interaction during the course of the tests in a large cohort (*n* = 117) of unimplanted control and defeated mice, stratified as “resilient” or “susceptible” as previously reported (Krishnan et al., 2007; Golden et al., 2011; Challis et al., 2013). Examining the cumulative time spent in the social interaction and corner zones we found that the behavior of susceptible mice significantly diverged from control and resilient as early as 4 s into the test (Repeated measures ANOVA, defeat × time, *F*_(298, 15049)_ = 49.894, *p* < 0.001; Figure 7A). Many mice in the latter two groups entered the social interaction zone immediately, with almost all entering under 40 s (Figure 7B), and continued to investigate the social target throughout the entire duration of the trial such that average interaction time accrued quasi-linearly in these groups (Linear regression, slope in cumulative time in seconds/second elapsed = 0.464 ± 0.005 for control, 0.4558 ± 0.004 for resilient, *r*^2^ = 0.741 for control, 0.616 for resilient; Figure 7A). In contrast, susceptible mice considerably delayed their decision to first enter the social interaction zone compared to resilient mice (under 50% had entered by 40 s; Kolmogorov-Smirnov test, *p* < 0.001; Figure 7B) and rarely returned to interaction zone after their first entry (Linear regression, slope = 0.1482 ± 0.002, *r*^2^ = 0.436 for susceptible; Figure 7A). Together, we interpret these data as an indication that the interindividual variability during the social interaction test reflects the execution of a binary choice between two behavioral strategies made a few seconds after the initiation of the task.

We applied the same time-course analysis to the dataset obtained from undefeated control mice receiving chronic photostimulation of vmPFC terminals during sensory exposure to novel aggressor mice. We found that the behavioral profile of undefeated mice that were implanted, but not stimulated, followed the same behavioral approach pattern as unimplanted control mice described above (Figure 7C). However, in striking contrast, undefeated mice whose vmPFC axon terminals in the DRN were photoactivated chose to remain in the distal corners from the beginning of the test [Repeated measures ANOVA, stimulation × time, *F*_(149, 894)_ = 16.18, *p* < 0.001; Figure 7D] and delayed their exploration of the novel social target for the majority of the trial [Repeated measures ANOVA, stimulation × time, *F*_(149, 894)_ = 6.79, *p* < 0.001; Figure 7C]. These mice also did not return to the social interaction zone as indicated by the plateau from 60 to 120 s (Linear regression, slope in cumulative time in seconds/seconds elapsed = 0.0196 ± 0.015, *r*^2^ = 0.007 for Laser group from 60 to 120 s). These results together suggest that enhancing glutamatergic drive from vmPFC axons in the DRN, in the presence of neutral social cues, functions as an aversive compound cue that bias subsequent choice toward an avoidance strategy.

## Discussion

Our results show that brief daily *ChR2*-mediated photoactivation of vmPFC inputs to the DRN temporally paired with sensory exposure to social cues in the absence of physical aggression resulted in a subsequent social avoidance phenotype, resembling that induced by social defeat. In addition, *Arch*-mediated photoinhibition of vmPFC inputs to the DRN during sensory contact phase in mice subjected to CSDS prevented the acquisition of social avoidance. Based on these results, we conclude that glutamatergic transmission within the vmPFC-DRN pathway bidirectionally modulates the valence perception of social cues. By characterizing the functional organization of DRN microcircuits underlying these biases, our results help clarify how maladaptive neuroplasticity of the vmPFC-DRN pathway could contribute to socio-emotional symptoms of affective disorders. These results also help conceptualize how somatic treatments such as DBS that target the vmPFC, may restore affective balance, partly through restoring neuroplasticty within the vmPFC-DRN pathway and altering DRN neurocircuitry (Veerakumar et al., 2013).

### Top-down drive of 5-HT output may be gated by DRN GABAergic neurons

The DRN is considered the primary nucleus containing forebrain-projecting 5-HT neurons, however 5-HT neurons account for less than half of the total neuronal population (Bang and Commons, 2012; Bang et al., 2012). One major non-serotonergic cellular population in this region is comprised of GABAergic neurons and we have previously shown that *GAD2*^+^ GABAergic neurons are the primarily activated neuronal population in the DRN in response to CSDS (Challis et al., 2013). In this work we show that axonal projections from the vmPFC localized in circumscribed subregions of the DRN that we found to be rich in defeat-sensitized *GAD2*^+^ cell bodies. Using whole-cell recording and *cF*os mapping after direct photoactivation of vmPFC terminals we determined that DRN GABA neurons were the direct and preferential synaptic targets of vmPFC projections in the DRN. In line with this hypothesized organization, an electrophysiological postsynaptic response in 5-HT neurons after vmPFC terminal photostimulation was not observed, however our experiments only sampled a limited population of neurons in the DRN using voltage clamp holding potentials standard for DRN 5-HT neurons (Beck et al., 2004). Recent work has also described the heterogeneity in physiological properties of 5-HT neurons in different DRN subfields that have known projections to dissimilar forebrain regions that regulate different types of behavior (Calizo et al., 2011; Crawford et al., 2011). Therefore, it will be important to probe a greater number of 5-HT neurons using various physiological conditions appropriate for the designated DRN subfield. We did observe a modest induction of *cFos* in 5-HT neurons after sustained photoactivation of vmPFC terminals, suggesting that direct excitatory influence from the vmPFC onto 5-HT neurons may exist, although the possibility that this may reflect an indirect effect cannot be excluded. The much greater percent of *cFos* induction observed in GABA neurons, though, suggests either a higher number of vmPFC projections targeting GABAergic neurons or an enhanced synaptic strength between vmPFC afferents and DRN GABA neurons.

In agreement with the findings here, previous studies had suggested preferential innervation of DRN GABA neurons by vmPFC terminals (Celada et al., 2001; Jankowski and Sesack, 2004; Hajós et al., 2007). Given these reports and our previous work showing that DRN GABA neurons locally synapse on and inhibit 5-HT neurons (Challis et al., 2013), we are presented with a putative circuit whereby DRN GABA neurons are positioned critically to gate top-down drive of the DRN and 5-HT output that would subsequently influence affective regulation. These same DRN GABA neurons have previously been shown to receive converging inputs from both the vmPFC and lateral habenula (Varga et al., 2003) and also possibly from CRF containing neurons originating in the amygdala and BNST (Waselus et al., 2005). It will be important in future experiments to determine whether the functional impact of vmPFC is dependent upon the coincident activity of these other inputs.

### Social valence choices are modulated by top-down projections from the vmPFC to the DRN

The vmPFC is classically thought of as an integrative hub that coordinates cognitive, affective, and autonomic dimensions of negative emotional experiences through distributed descending inputs to subcortical regions in the limbic system and brainstem (Roy et al., 2012). Animal studies suggest this role partly involves top-down modulation of 5-HT neurons in the brainstem raphe nuclei. Multiple studies relying on pharmacological inactivation methods (Amat et al., 2005, 2006; Christianson et al., 2009; Slattery et al., 2011), electrical stimulation (Hamani et al., 2010, 2011; Veerakumar et al., 2013) or optogenetics (Warden et al., 2012; Kumar et al., 2013) have implicated vmPFC-DRN circuits in the regulation of behavioral response to aversive challenges. However, based on the data currently available there is not a consensus as to whether activation of cortical inputs in the DRN inhibits or promotes 5-HT output. It is also controversial whether this then mediates aversion or on the contrary, facilitates anti-aversive responses. Although there is solid evidence that electrical stimulation of the vmPFC inhibits the firing of 5-HT cells, concurrent measures of extracellular 5-HT using microdialysis have also reported corresponding enhancements of extracellular 5-HT in the DRN and forebrain (Celada et al., 2001; Hamani et al., 2010), further complicating the relationship between firing and release.

Recent computational models have posited that 5-HT codes for threat prediction signals, particularly during tasks that use behavioral inhibition as a readout for aversion in human and animals (Soubrie, 1986; Crockett et al., 2012). Reduction in tonic 5-HT levels after tryptophan depletion (presumably resulting in a gain in signal-to-noise for 5-HT phasic signals, see Cools et al., 2008) has been associated with enhanced neural processing and detection of social threats (Harmer, 2012; Passamonti et al., 2012), punishment prediction (Cools et al., 2008), and increased social defensiveness (Young, 2013). Importantly, avoidance biases in response to ambiguous social cues are reported in patients suffering from depression and social phobia (Heuer et al., 2007; Seidel et al., 2010; Derntl et al., 2011; Volman et al., 2011; Moser et al., 2012). In the social interaction task we used here, mice confronting an “ambiguous” social target resembling their aggressor made a rapid binary choice between two alternative behavioral strategies (e.g., active risk assessment through social approach or social avoidance by remaining in a distal corner). Our results show that susceptible mice have a bias toward avoidance that resemble responses of depressed patients in laboratory approach-avoidance tasks. Whether this choice is effectively determined by modulation of 5-HT levels remains to be determined, however, in the context of social interactions, dishinibition of 5-HT neurons via pharmacological autoinhibition of GABA neurons in the DRN, which increases 5-HT output in the forebrain regions such as the vmPFC, has consistently been shown to promote social approach and offensive behaviors of defeated mice (Takahashi et al., 2010, 2012). This is in general agreement with data linking enhancement of forebrain 5-HT output with resilience to social stress and maintenance of dominant social status in various species (Raleigh et al., 1991; Malatynska et al., 2005; Alekseyenko et al., 2010; Penn et al., 2010; Bruchas et al., 2011) and thus, DRN GABA neurons may pose as a key cellular population in mediating social choice through regulation of 5-HT output.

Our results demonstrate that chronically activating vmPFC inputs in the DRN is behaviorally pro-depressive, however they are at odds with results from Warden et al who reported time-locked antidepressant-like effects in the forced swim test (FST) upon direct, acute optogenetic activation of vmPFC glutamatergic terminals in the DRN (Warden et al., 2012). Our results are also difficult to reconcile with the model proposed by Maier and colleagues, whereby vmPFC-driven reductions in DRN 5-HT output mediate resistance to learned helplessness in rats (Amat et al., 2005). The apparent contradictions between these studies (reviewed in detail by Lammel et al., 2014) could derive from obvious differences in the models employed, with the most likely being the different defense systems (behavioral inhibition vs. flight) that are engaged during these tasks and the contradictory regulation by 5-HT (Deakin and Graeff, 1991). Nevertheless, our results clearly establish a key role of vmPFC afferents to the DRN in biasing approach-avoidance choices and begin to lay the groundwork for a mechanism of regulating 5-HT output in processing underlying affective resilience.

### Conflict of interest statement

The authors declare that the research was conducted in the absence of any commercial or financial relationships that could be construed as a potential conflict of interest.
